# Alcohol use in Tanzanians with chronic psychotic disorders and poor medication adherence

**DOI:** 10.4102/sajpsychiatry.v27i0.1570

**Published:** 2021-03-19

**Authors:** Emily Simon, Jennifer B. Levin, Jessie Mbwambo, Carol Blixen, Isaac Lema, Michelle Aebi, Godwin Njiro, Kristin Cassidy, Sylvia Kaaya, Martha Sajatovic

**Affiliations:** 1 School of Medicine, Case Western Reserve University, Cleveland, The United States of America; 2 Department of Psychiatry, School of Medicine, Case Western Reserve University, Cleveland, The United States of America; 3 Neurological & Behavioral Outcomes Center, School of Medicine, Case Western Reserve University, Cleveland, The United States of America; 4 Department of Psychiatry and Mental Health, School of Medicine, Muhimbili University of Health and Allied Sciences, Dar es Salaam, Republic of Tanzania; 5 University Hospitals Cleveland Medical Center, Cleveland, The United States of America; 6 Department of Neurology, School of Medicine, Case Western Reserve University, Cleveland, The United States of America

**Keywords:** schizophrenia, medication adherence, treatment adherence, alcohol abuse, AUDIT, ASSIST, chronic psychotic disorders, sub-Saharan Africa, substance abuse

## Abstract

**Background:**

The burden of chronic psychotic disorders (CPDs) in sub-Saharan Africa (SSA) is significant. Poorly medically adherent patients are more likely to have worse outcomes and require more resources. However, factors impacting effective treatment of CPD in this population are unclear.

**Aim:**

Examine the relationship between alcohol use and disease management and compare alcohol risk stratification between the Alcohol Use Disorders Identification Test (AUDIT) and Alcohol, Smoking and Substance Involvement Screening Test (ASSIST) in poorly medication adherent Tanzanians with CPD.

**Setting:**

Muhimbili National Hospital and ambulatory clinics in Dar es Salaam, Tanzania.

**Methods:**

100 Tanzanians with CPDs and suboptimal medication adherence were dichotomized into low and moderate-to-high risk alcohol use based on AUDIT scores and compared regarding medication attitudes, adherence and psychiatric symptoms. Patients completed the ASSIST for comparison to AUDIT risk stratification.

**Results:**

Moderate-to-high risk alcohol users had worse medication attitudes (*p* < 0.01), medication adherence (previous week, *p* = 0.01; previous month, *p* < 0.001), and psychiatric symptoms (*p* = 0.03). They were younger, predominately male and more likely to have a family history of alcohol abuse. A logistic regression analysis found age, gender and family history of abuse as significant predictors of hazardous alcohol use (*p* = 0.02, 0.02, < 0.01, respectively). Risk stratification between AUDIT and ASSIST aligned in 85% of participants.

**Conclusion:**

Alcohol use is an important consideration in treating poorly adherent Tanzanians with CPD. The ASSIST was comparable to the AUDIT in stratifying risky alcohol use with the additional benefit of screening for other substances.

## Introduction

Chronic psychotic disorders (CPDs) such as schizophrenia and schizoaffective disorder can pose significant burden to affected individuals^1,2^ and the communities where they reside.^3,4^ Reduced quality of life, disorder relapse, impaired personal and professional achievement and premature mortality because of suicide or other causes characterise some of the challenges that can arise when treatment is insufficient.^2,5^ Concurrent psychosocial services and antipsychotic medications along with appropriate monitoring of health status are the mainstay of disease management.^6^ However, access and proper utilisation of mental healthcare services are a challenge globally, with estimations as dismal as 76% – 85% of patients in low- and middle-income countries going without treatment.^2^ Indeed, predictions indicate that mental health resources will be in continued deficit in coming years, amplified by growing disease burden.^7^ For example, in sub-Saharan Africa (SSA), the most disabling conditions amongst those aged 10–14 years are brain disorders.^8,9^ This includes regions such as Tanzania, where neuropsychiatric disorders are estimated to make up approximately 5.3% of the global burden of disease.^10^

In SSA, poor medication adherence is seen in approximately half of those with CPD and is a major driver of relapse.^11,12,13,14^ In addressing adherence, consideration must be made about factors influencing access and attitudes towards medications in this region. One potential factor is alcohol use.^15^ Many epidemiologic studies worldwide have demonstrated elevated prevalence of substance use disorders in individuals with schizophrenia compared with the general population.^16,17,18,19^ Furthermore, substance abuse contributes to poor medication adherence in those with schizophrenia^15^ and is a predictor of poor outcomes, including premature death, in psychiatric patients.^20^ Amongst the substances abused, alcohol has been cited as one of the most prevalent, often exceeded only by nicotine.^17,21^

Most of the available data, however, have been obtained from western countries. Less is known about the relationship between alcohol use and schizophrenia in regions of the world such as SSA where the culture around and availability of alcohol differs from that of North American or European countries. Alcohol use in SSA varies substantially based on the diversity of ethnicities, religion acceptability and political and economic stability.^22,23,24^ Although limited in number, studies conducted in SSA countries such as Kenya, Zimbabwe and Nigeria indicate that alcohol abuse is prevalent in those with CPD, suggesting a similar pattern to that seen globally.^12,25,26,27,28^

In Tanzania, substance use appears to be a growing problem. Although country-wide prevalence is less than in other affluent countries, substance use disproportionally impacts those living in poorer areas,^29^ with tobacco products and alcohol being the most widely used.^30^ In a study specific to Tanzanian psychiatric patients, this pattern remained. Alcohol and tobacco were the most commonly used substances (59.3% and 38.6%, respectively), with significantly higher rates of use in men than in women.^31,32^ In addition, the use of cannabis, inhalants, amphetamines and sedatives was observed, albeit in a smaller proportion of patients.

Alcohol use specific to Tanzanians with CPD appears absent in the current body of literature, warranting further exploration of this unique patient population. In addition, amongst individuals with CPD, research assessments can be burdensome given that the conditions impede concentration and one’s ability to participate in more lengthy interviews. Thus, comparing the responses and characteristics of alcohol use captured by two frequently used relatively brief questionnaires, the Alcohol Use Disorders Identification Test (AUDIT) and the Alcohol, Smoking and Substance Involvement Screening Test (ASSIST), in this specific patient population could streamline the interview process and further validate the use of these measures in the unique sociocultural context of Tanzania. This study examines the relationship between patterns of alcohol use and patient demographics, medication attitudes, adherence and psychiatric symptoms in Tanzanians with CPD. In addition, it provides a comparison of the AUDIT and ASSIST, discerning how the measures complement each other and assess different or overlapping aspects of alcohol use in this population.

## Methods

### Overview

The data for this cross-sectional study were collected as part of a larger 3-phase/3-aim 24-month project focused on medication adherence enhancement in Tanzanians with CPDs and a history of poor medication adherence. The first phase of the study consisted of an observational mixed-methods (quantitative and qualitative) approach aimed at assessing reasons for poor treatment adherence amongst Tanzanians with CPD. The data and analysis described in this article were derived from the phase 1 quantitative assessment, collected between April and December of 2018. All study assessments and questions asked to patients were translated into Swahili and collected by research assistants trained by the study’s principal investigators. A more complete methodological overview of the project is described elsewhere.^33^

### Participants

A total of 100 individuals with CPD were recruited from Muhimbili National Hospital and its associated ambulatory clinics, a number estimated to represent 20% of individuals with CPD in that clinical setting at the time. Those who self-reported missing 20% or more of their antipsychotic medication within the last month as measured by the Tablets Routine Questionnaire (TRQ),^34^ an established benchmark for poor adherence,^35,36^ were eligible to participate. Patients were of 18 years or older with a clinical diagnosis of schizophrenia or schizoaffective disorder who had recently been hospitalised because of relapse of disease symptoms related to sub-optimal treatment adherence. Exclusion criteria included history of allergy or intolerance to haloperidol or haloperidol decanoate, immediate prior use of long-acting injectable antipsychotic medication, medical condition or illness that would interfere with the patient’s ability to participate (determined by research psychiatrist), physical dependence on substances likely to lead to withdrawal reaction during the study course, immediate risk of harm to self or others, pregnancy or breastfeeding. Written informed consent was obtained from all the participants and the study was approved by the local Institutional Review Board (IRB).

### Alcohol Use Disorders Identification Test and Alcohol, Smoking and Substance Involvement Screening Test assessment, scoring and indications

Substance use was measured using the AUDIT^37^ and ASSIST^38^ tests. Both measures were developed by the World Health Organization (WHO) and have been validated in settings globally.^39,40^

Since the original development, the AUDIT has become one of the most heavily studied and widely used screening measures for risky alcohol consumption,^41,42^ with sensitivity in the mid-0.90s and specificity around 0.80s in the test development samples.^39^ The AUDIT consists of 10-items specific to alcohol use. The questions are categorised by the WHO as capturing three domains of drinking behaviours: hazardous alcohol use (frequency and quantity), symptoms of alcohol dependence (control over drinking, prominence of behaviour and morning consumption) and harmful alcohol use (emotional impact of drinking, memory loss, injuries and others’ concern).

Scores of 8 or more are considered indicators of hazardous (8–15), harmful (16–19), high risk (20 or more) and/or dependent alcohol use, with a total possible score of 40. The categories provide indications for varying levels of intervention, such as simple advice (hazardous, score of 8–15), brief counselling and continued monitoring (harmful, score of 16–19) or referral to a specialist for diagnostic evaluation and treatment (high risk, score of 20–40). Alcohol dependency is scored independently by adding up the scores of questions 4–6, with a score of 4 or more (maximum score = 12) indicating the need to assess for dependency, even if in a lower risk category.^39^

The 8-item ASSIST evaluates a range of substances, including alcohol, tobacco, cannabis, cocaine, amphetamines, inhalants, sedatives and hallucinogens. The first question assesses whether a respondent has ever used a given substance using a binary ‘yes/no’ response. Responses of ‘no’ terminate further questioning about that particular substance. Questions 2–7 assess frequency, craving, use-related problems, others’ concern and control over use. The questions are repeated for any substance a respondent indicated as having used previously in question 1. The final question asks whether the participant has ever used any substance via injection.

Each substance is scored individually. For alcohol, scores of 0–10 indicate low risk and no need for intervention, scores of 11–26 indicate moderate risk and receiving a brief intervention and scores of 27 indicate high risk and the need for more intensive treatment. All other substances are divided into similar categories of low, moderate and high risk, but with cut-off scores of 0–3, 4–26 and 27 or more, respectively.^40^

### Additional data collection

Information on demographic and clinical characteristics relevant to CPD relapse was collected using several previously validated measures. The degree of adherence was assessed using the TRQ,^34^ which captures the proportion of days with medication doses missed in the past 1 week and 1 month. The questions are answered for each of a patient’s prescribed medications. A higher score indicates worse adherence.^34^

In order to assess the barriers to adherence and attitudes towards medication, participants completed the Rating of Medication Influences (ROMI),^43^ the Attitudes towards Mood Stabilisers Questionnaire (AMSQ),^44,45^ and the Drug Attitude Inventory (DAI).^46^ The ROMI was originally developed for populations with schizophrenia, in which it was found to be reliable, clinically sound and valid when compared with other independent measures of attitudes and adherence.^43^ For the current project, only Part II of the questionnaire was administered, which is comprised of 10 items that ask directly about influences leading to non-adherence (including how medications make one feel, medications’ effect on goals and appearance to others, support system’s opinion on medications, access to medications). The AMSQ is a modification of the Lithium Attitudes Questionnaire^45^ that evaluates attitudes towards psychiatric medication, consisting of 19 items grouped into 7 subscales (general opposition to prophylaxis [4 items], denial of therapeutic effectiveness [2 items], fear of side effects [2 items], difficulty with medication routines [4 items], denial of illness severity [3 items], negative attitudes towards drugs in general [3 items] and lack of information about psychiatric medication [1 item]). Higher scores indicate more negative attitudes towards mood stabilisers. Recent work found the AMSQ to be valid (with test–retest reliability of *p* = 0.73) and sensitive to changes in time as evidenced by changing scores correlating with changes in the TRQ.^47^ Finally, the DAI was used to assess attitudes towards medication in individuals with serious mental illness, consisting of 10 items in a ‘true or false’ format.^46^ It has been shown to be relatively unaffected by psychiatric symptom severity^48^ and is scored via a continuum, ranging from –10 to +10, with higher scores indicating better attitudes. Reliability was determined by the developers to be 0.93.^49^

Chronic psychotic disorders symptoms were assessed using the Brief Psychiatric Rating Scale (BPRS),^50^ which consists of 18 items scored on a 1–7 scale. It assesses both the major psychotic and non-psychotic symptoms in those with serious mental illnesses. The measure has been widely researched and validated, with reliability coefficients reported to be between 0.63 and 0.87.^49^

Global psychopathology was measured using the Clinical Global Impressions (CGI) scale,^51^ which evaluates illness severity on a 1–7 point scale. Reported reliability scores for severity illness range from 0.41 to 0.60.^49^

Life and work functional status were evaluated using the Social and Occupational Functioning Assessment Scale (SOFAS).^52^ The SOFAS is derived from the Global Assessment of Functioning (GAF), which consists of a 100-point single-item scale that measures global functioning of psychiatric patients. Lower scores indicate lower functioning. It has been shown to be reliable and valid in studies involving patients with serious mental illness,^53^ with reliability coefficients ranging from 0.62 to 0.82.^49^

### Alcohol abuse risk dichotomisation and statistical analysis

Participants were dichotomised into groups based on AUDIT scores. Participants who scored 7 or less were deemed ‘low risk’, whilst those who scored 8 or more were deemed ‘moderate to high risk’. Given the small number of participants with scores 8 and above, all such participants were merged into a single group. These risk groups were compared on attitudes towards medication, medication adherence, psychiatric symptoms and demographics. Comparisons were made using two-tailed *t*-tests for normally distributed variables and Mann–Whitney U for non-normal TRQ distribution. A chi-square test was used for comparisons of tobacco and cannabis use between dichotomised alcohol risk groups.

### Ethical considerations

The UH IRB operates under HHS Federalwide Assurance (FWA) number 00003937 and IRB registration numbers 00000684, 00001691 and 00008600. The CWRU IRB operates under DHHS FWA00004428 and IRB registration number 00000683.

## Results

### Alcohol use risk groups

Table 1 reflects the AUDIT risk categorisation based on the WHO scoring guidelines. Of the 100 participants, 72 scored 7 and below (defining the study’s ‘low risk’ participants), whilst 28 scored 8 and above (defining the study’s ‘moderate to high risk’ participants). Of those in the latter group, nine participants scored as ‘almost certainly dependent on alcohol’ in the dependency subscale.

**TABLE 1:** Alcohol abuse risk defined by the Alcohol Use Disorders Identification Test in Tanzanians with chronic psychotic disorder and suboptimal medication adherence.

AUDIT	*n*	%	SD
Mean	5.38	-	8.03
Median	0.00*	-	-
Range	0 to 31	-	-
**AUDIT total score**			
0 – 7	72	72.0	-
8 – 15	15	15.0	-
16 – 19	4	4.0	-
20 or more	9	9.0	-
Almost certainly dependent on alcohol	9	9.0	-

AUDIT, Alcohol Use Disorders Identification Test.

* , A total 52 participants reported no lifetime use of alcohol, scoring a zero on the AUDIT.

### Demographics

Table 2 presents the analysis of demographics by alcohol use group. No statistically significant differences were observed between low risk versus moderate to high risk participants in marital status, education level or employment status. The ages of the moderate to high risk users were significantly younger than the low risk participants (32.39 vs. 36.99 years, respectively; *p* = 0.02) and moderate to high risk users were less likely to have children than low risk participants (39.3% vs. 63.9%, respectively; *p* = 0.04). Furthermore, a significantly higher proportion of moderate to high risk users were male as compared to female (*p* < 0.01).

**TABLE 2:** Demographic and clinical characteristics dichotimised by Alcohol Use Disorders Identification Test-defined low risk and moderate to high risk alcohol use groups.

Variable	Mean (SD) or *N* (%) *N* = 100				Low risk *N* = 72			Moderate to high risk *N* = 28			Statistic†
	Mean or *N*	SD or %	Median	Range	Mean or *N*	SD or %	Median	Mean or *N*	SD or %	Median	
**Age**	35.70	8.80	-	19 to 62	36.99	8.81	-	32.39	8.01	-	***t*(98)= 2.40, *p* = 0.02**
**Gender**											**χ^2^(1) = 9.99, *p* < 0.01**
Male	61	61.0	-	-	37	51.4	-	24	85.7	-	
Female	39	39.0	-	-	35	48.6	-	4	14.3	-	
**Marital Status**											χ^2^(2) = 2.08, *p* = 0.35
Single, never married	58	58.0	-	-	39	54.2	-	19	67.9	-	
Married	28	28.0	-	-	21	29.2	-	7	25.0	-	
Separated /divorced/widowed	14	14.0	-	-	12	16.7	-	2	7.1	-	
**Children (*n* = 99)**											**χ^2^(1) = 4.31, *p* = 0.04**
Have children	57	57.0	-	-	46	63.9	-	11	39.3	-	
Do not have children	42	42.0	-	-	26	36.1	-	16	57.1	-	
**Number of children (*n* = 99)**	1.39	1.81	1	0 to 10	1.67	1.98	-	0.67 (*n* = 27)	0.96	-	***U* = 629.50, *p* = 0.005**
**Education level – years (*n* = 99)**	9.42	3.61	-	2 to 19	9.76 (*n* = 71)	3.67	-	8.57	3.37	-	*t*(97) = 1.48, *p* = 0.14
**Employment**											χ^2^(2) = 1.92, *p* = 0.38
Full time	23	23.0	-	-	19	26.4	-	4	14.3	-	
Part time	29	29.0	-	-	19	26.4	-	10	35.7	-	
Unemployed	48	48.0	-	-	34	47.2	-	14	50.0	-	
**Primary residence**											χ^2^(2) = 0.63, *p* = 0.73
Lives alone	11	11.0	-	-	9	12.5	-	2	7.1	-	
Lives with family	85	85.0	-	-	60	83.3	-	25	89.3	-	
Other^b^	4	4.0	-	-	3	4.2	-	1	3.6	-	
**Diagnosis**							-				χ^2^(1) = 0.11, *p* = 0.74
Schizophrenia	80	80.0	-	-	57	79.2	-	23	82.1	-	
Schizoaffective	20	20.0	-	-	15	20.8	-	5	17.9	-	
**Age of CPD onset**	22.38	7.64	-	1 to 49	23.15	8.02	-	20.39	6.25	-	*t*(98) = 1.64, *p* = 0.11
**Illness duration – years**	12.39	7.99	-	1 to 36	12.76	8.16	-	11.43	7.60	-	*t*(98)= 0.75, *p* = 0.46
**Treatment status at assessment**											χ^2^(1) = 1.09, *p* = 0.30
Inpatient	83	83.0	-	-	58	80.6	-	25	89.3	-	
Outpatient	17	17.0	-	-	14	19.4	-	3	10.7	-	
**CPD medication prescribed**											χ^2^(4) = 1.61, *p* = 0.81
Chlorpromazine	8	8.0	-	-	6	8.3	-	2	7.1	-	
Haloperidol	72	72.0	-	-	50	69.4	-	22	78.6	-	
Olanzapine	11	11.0	-	-	8	11.1	-	3	10.7	-	
Risperidone	8	8.0	-	-	7	9.7	-	1	3.6	-	
Trifluoperazine	1	1.0	-	-	1	1.4	-	0	0.0	-	
**Lifetime hospitalisations**											
Psychiatric	4.72	4.30	3.00	-	4.60	4.13	3.00	5.04	4.74	3.50	*U* = 0.37, *p* = 0.71
Substance abuse	0.45	1.35	0.00	-	0.29	0.85	0.00	0.86	2.14	0.00	*U* = 1.40, *p* = 0.16
**History of Abuse**											
Physical (*n* = 99)						-					χ^2^(1) = 0.42, *p* = 0.52
Yes	55	55.0	-	-	38	52.8	-	17	60.7	-	
No	44	44.0	-	-	33	45.8	-	11	39.3	-	
Sexual											χ^2^(1) = 0.08, *p* = 0.78
Yes	27	27.0	-	-	20	20.8	-	7	25.0	-	
No	73	73.0	-	-	52	72.2	-	21	75.0	-
**Family history**											
Mental illness											χ^2^(1) = 1.99, *p* = 0.16
Yes	53	53.0	-	-	35	48.6	-	18	64.3	-	
No	47	47.0	-	-	37	51.4	-	10	35.7	-	
Alcohol abuse											**χ^2^(1) = 7.19, *p* < 0.01**
Yes	43	43.0	-	-	25	34.7	-	18	64.3	-	
No	57	57.0	-	-	47	65.3	-	10	35.7	-

Bold values indicate statistical significance.

CPD, chronic psychotic disorder.

† , Two-tailed *t*-test, chi-square comparison, or Mann–Whitney *U* test for non-parametric data between low and moderate drinking risk;

‡ , Two participants lived with people other than family, and two participants lived in rehabilitation centres.

Where *n* does not equal 100, a participant failed to complete the given measure properly or entirely.

With regard to CPD variables, there was no significant difference in the age of onset, illness duration, medication prescribed or lifetime hospitalisations. Although family history of mental illness did not significantly differ between groups, there was a significant difference in family history of alcohol abuse, with 64.3% of moderate to high risk participants reporting a family history of alcohol abuse as opposed to 34.7% of low-risk participants (*p* < 0.01).

### Medication adherence, psychiatric symptoms and attitudes towards medication

Table 3 presents the scores on various questionnaires. Those with moderate to high risk had significantly higher scores on the TRQ, indicating a greater percentage of days in which at least one dose of medication was missed, in both the past 1 week and the past 1 month (*p* = 0.01 and *p* < 0.001, respectively). Moderate to high risk users also scored significantly higher on the AMSQ and ROMI, both of which indicate more negative attitudes towards medication (*p* < 0.01 and *p* < 0.01, respectively). On the BPRS, reflecting the severity of psychiatric symptoms, moderate to high risk users scored significantly higher (*p* < 0.05). No significant difference was observed on the CGI, SOFAS or DAI.

**TABLE 3:** Questionnaire responses dichotimised by Alcohol Use Disorders Identification Test-defined low risk and moderate to high risk alcohol use groups.

Measures	Low risk (*N* = 72)		Moderate to high risk (*N* = 28)		*t*-test/Mann–Whitney U*
	*n*	%	*n*	%	
TRQ†*					
Past week	86.92	23.68	97.46	13.42	***U* = 771.50, *p* = 0.01**
Past month	56.08	37.53	85.93	28.77	***U* = 573, *p* < 0.001**
**CGI**‡					
Severity	2.49	0.93	2.61	1.07	*t*(98) = −0.56, *p* = 0.58
Global improvement	1.63	1.03	1.86	0.76	*t*(66.62) = −1.24, *p* = 0.22
BPRS§	33.51	8.01	37.54	8.23	***t*(98) = −2.24, *p* = 0.03**
SOFAS¶	65.35	9.43	61.39	8.24	*t*(98) = 1.95, *p* = 0.054
DAI††	6.68	1.80	6.71	1.74	*t*(98) = −0.09, *p* = 0.93
AMSQ‡‡ (*n* = 99)	7.37, *n* = 71	3.36	9.50	3.72	***t*(97) = −2.76, *p* < 0.01**
ROMI§§ (*n* = 98)	13.19, *n* = 70	4.23	16.39	5.51	***t*(96) = −3.10, *p* < 0.01**

Bolded values indicate statistical significance.

† , TRQ: Tablet Routines Questionnaire: Percentage of days in which at least one dose of medication was missed.

‡ , CGI: Clinical Global Impression; higher scores indicate more severe illness.

§ , BPRS: Brief Psychiatric Rating Scale: Higher scores indicate more severe symptoms.

¶ , SOFAS: Social and Occupational Functioning Assessment Scale; higher scores indicate a high level of functioning.

†† , DAI: Drug Attitudes Inventory: Higher scores indicate better drug attitudes.

‡‡ , AMSQ: Attitudes towards Mood Stabilisers Questionnaire: Higher scores indicate worse medication attitudes.

§§ , ROMI: Rating of Medication Influence: Lower scores indicate more positive attitudes, whilst higher scores indicate more negative attitudes.

* , indicates use of Mann-Whitney as opposed to a *t*-test.

When separated by gender, moderate to high risk men had significantly higher scores on the TRQ for adherence over the past 1 month and on the AMSQ than low-risk men, but all other measures did not show any statistical difference. Given that so few women were in the moderate to high risk category (*n* = 4), further interpretation was not possible for women (data not shown).

### Logistic regression model

A logistic regression model was performed with eight variables used to predict moderate to high risk alcohol use. The variables selected were significantly correlated with moderate to high risk alcohol use on bivariate analysis and included age, gender, having children, family history of alcohol abuse, medication adherence over the past month (as determined by the TRQ), psychiatric symptoms (as determined by BPRS) and two measures of medication attitudes (AMSQ and ROMI). The model fit the data well (omnibus test of model coefficients, chi-square = 41.50, degrees of freedom = 8, *p* < 0.001; Nagelkerke R-square = 0.502; Hosmer and Lemeshow test, chi-square= 10.59, degrees of freedom = 8, *p* = 0.226) and correctly classified 83.5% of study participants.

Age, gender and family history of abuse were significant predictors. According to the model, for every increase in age by 1 year, individuals have a 9.7% decrease in odds of moderate to high risk alcohol use (odds ratio [OR] = 0.903; 95% confidence interval [CI], 0.83–0.99; *p* = 0.02). Men are 7.01 times more likely than women to have a moderate to high risk alcohol use (OR = 7.01, 95% CI, 1.46–33.82, *p* = 0.02). Individuals with a family history of alcohol abuse are 6.46 times more likely to have a moderate to high risk alcohol use (OR = 6.46, 95% CI, 1.77–23.59, *p* < 0.01). All other predictors were not significant.

### Additional substance use

Table 4 presents reported substance use on the ASSIST. Alcohol, tobacco, and cannabis had the highest risk use rates (moderate to high risk users).

**TABLE 4:** Breakdown of Alcohol, Smoking and Substance Involvement Screening Test-defined substance abuse risk.

Risk category	Tobacco (*n* = 100)	Alcohol (*n* = 100)	Cannabis (*n* = 100)	Cocaine (*n* = 100)	Amphetamine (*n* = 100)	Inhalants (*n* = 100)	Sedatives (*n* = 100)	Hallucinogens (*n* = 100)	Opioids (*n* = 100)	Other (*n* = 100)
Low risk†	65	68	84	100	100	99	99	100	100	100
Moderate risk	32	25	15	0	0	1	1	0	0	0
High risk	3	7	1	0	0	0	0	0	0	0

† , Low-risk category includes participants who reported no lifetime use of a given substance.

Table 5 presents the tobacco and cannabis use compared between low and moderate to high risk drinkers. Those with moderate to high risk alcohol consumption reported significantly higher rates of both tobacco and cannabis use on the ASSIST when compared with the low-risk participants (*p* < 0.01 and *p* < 0.001, respectively; Table 5).

**TABLE 5:** Tobacco and cannabis use dichotimised by Alcohol Use Disorders Identification Test-defined low risk and moderate to high risk alcohol use groups.

	Low risk, *n* = 72		Moderate to high risk, *n* = 28		Chi-square
	*n*	%	*n*	%	
**Smokes tobacco?**					
No	35	48.6	5	17.9	***χ*^2^(1) = 7.95, *p* < 0.01**
Yes	37	51.4	23	82.1	
**Uses cannabis?**					
No	49	68.1	8	28.6	***χ*^2^(1) = 12.82, *p* < 0.001**
Yes	23	31.9	20	71.4	

Bold values indicate statistical significance.

### Alcohol Use Disorders Identification Test versus Alcohol, Smoking and Substance Involvement Screening Test responses

Table 6 presents a comparison between the AUDIT and the alcohol-specific ASSIST responses. Of the 100 participants, 85 were assessed as having similar risk in drinking behaviour via both measures (i.e. scored to be in an equivalent risk categorisation). Of the 15 participants who were discrepant between the two measures, only one participant was discrepant by two risk categories, scoring high risk on the AUDIT but low risk on the ASSIST.

**TABLE 6:** Alcohol abuse risk categorisation on the Alcohol Use Disorders Identification Test versus the Alcohol, Smoking and Substance Involvement Screening Test.

ASSIST risk categories	AUDIT risk categories			Total ASSIST
	Low risk	Moderate risk	High and harmful risk	
Low risk	**67**	0	1	68
Moderate risk	5	**13**	7	25
High risk	0	2	**5**	7

**Total AUDIT**	72	15	13	100

Bolded values indicate non-discrepant participants (*n* = 85).

AUDIT, Alcohol Use Disorders Identification Test; ASSIST, Alcohol, Smoking and Substance Involvement Screening Test.

## Discussion

The results of this study suggest that alcohol use is an important consideration in the landscape of disease management for those with CPD and poor medication adherence in Tanzania. In 100 patients experiencing relapse of their disorder, over a quarter presented with appreciable risk in terms of alcohol consumption (28%) as defined by the AUDIT. Interestingly, of those who reported *any* alcohol use at all, that is, including low-risk drinkers, over half were assessed to be at moderate-to-high risk for abuse (58.3%). This may reflect the significant population of lifetime abstainers commonly seen in Tanzania,^39^ as 52 of the participants reported no alcohol use whatsoever (scoring a zero on the AUDIT), representing the majority of the low-risk participants. This has important clinical implications as the apparent prevalence of risky alcohol use may be masked by large numbers of lifetime abstainers. If the absolute prevalence impacts clinical screening practices, the significant minority of patients with alcohol use problems may be missed and the opportunity for appropriate interventions may be lost.

Evidence for this pitfall has been recorded elsewhere in SSA. In a study at Mathari Psychiatric Hospital in Kenya, of the 238 patients who met Diagnostic and Statistical Manual of Mental Disorders (DSM-IV) criteria for substance abuse disorder, only 49 had ‘substance use disorder’ as part of their current admission differential diagnosis. Furthermore, despite alcohol dependency being the most common Structured Clinical Interview for the DM (SCID) substance abuse disorder, the authors noted that this was rarely recorded in the hospital diagnosis and only 12 patients were receiving drug rehabilitative treatment.^25^

Demographically, moderate to high risk users differed significantly in terms of age, number of children, family history of substance abuse and gender. Of the significant bivariate differences, age, gender and family history of alcohol abuse were found to be significant predictors of moderate to high risk alcohol use (whilst the number of children was not) in our logistic regression model. The trend towards risky drinking in younger participants is congruent with recent work suggesting high rates of alcohol consumption in youth in Tanzania.^54,55^ The observed association between moderate to high risk alcohol consumption and family history of substance abuse is consistent with a large body of work suggesting family history as a predictor of substance abuse.^56,57^ Finally, male predominance in patients reporting moderate to high risk alcohol consumption is consistent with that observed in other populations throughout Tanzania,^55,58,59^ including the previous study involving Tanzanian psychiatric patients.^31^ Women with schizophrenia have been found to have lower rates of substance abuse across many diverse regions of the world.^60^

Risky alcohol use in those with CPD was associated with poorer medication adherence, worse attitudes towards medication (as measured by the AMSQ and ROMI) and greater severity of psychiatric symptoms. This aligns with other studies examining non-adherence to antipsychotic medications. In one systemic review, negative attitudes towards medication and substance abuse emerged as the most consistent correlates to poor adherence.^15^ Although the causality between these factors cannot be determined from this study, it is reasonable to assume that addressing risky alcohol use would be a logical addition to the treatment plan for those with CPD and poor adherence, particularly in light of the correlation with symptom severity.

The study’s findings are better appreciated when compared with the general Tanzanian population. The 2018 WHO report on alcohol estimated 47.1% of the population aged 15 years and older to be lifetime abstainers and 63.6% to have abstained from drinking alcohol over the past 1 year. This is consistent with this study population, where 63 (63%) participants reported ‘Never’ on the AUDIT to the question ‘How often do you have a drink containing alcohol?’ However, rates of alcohol use disorders (as defined by the WHO report as ‘including alcohol dependence and harmful use of alcohol’) and alcohol dependence were greater in the study population. A total of 13% of participants had ‘high-risk or harmful level’ or ‘dependent on alcohol’ categorisation via the AUDIT, compared with WHO estimates of 6.8% for alcohol use disorder in the general population. In addition, 9% of the study population were almost certainly dependent on alcohol, whilst only 2.4% of the general population were predicted to have alcohol dependency.^58^ Although such comparisons are crude, they provide an important sociocultural context. The trend that those with CPD have higher rates of substance abuse than the general public is consistent with that seen in westernised countries.^17^ However, without comparing the rates seen in Tanzanians with CPD against the background estimates of Tanzanians in general, this trend might be missed, as the absolute rates (13% and 9%) are much lower than those seen, for example, in the United States of America (one study reported, as a comparison, 33.7% of those diagnosed with schizophrenia or schizophreniform disorder to meet the criteria for alcohol disorder^17^). One might suspect that, based on the findings of the WHO report, the lower rates seen in Tanzanians with CPD in this study than those seen in westernised countries is a function of the underlying accessibility, normative use and disposable income driving alcohol consumption practices in a more underdeveloped country – however, more work is needed to determine that conclusively.

An important consideration in detecting substance abuse in any patient population (and particularly in vulnerable patients with CPD who struggle with medication adherence) is the efficiency and validity of the screening measure used. The ASSIST, similar to the AUDIT, was developed by the WHO as a response to the global burden of psychoactive substance use.^40^ Screening for risky substance use over a range of substances, including alcohol, it offers an opportunity to streamline the clinical assessment whilst capturing a more complete picture of substance use than the AUDIT. For example, tobacco and cannabis were also used by many patients and those with riskier alcohol consumption were more likely to smoke tobacco and use cannabis (Table 5).

This study showed overlap in the risk categorisation between the AUDIT and ASSIST (Table 6), suggesting that the ASSIST captures an individual’s alcohol risk similar to the AUDIT. Most of the participants had an equivalent risk stratification between the two measures (85%) and of the 15% who were incongruent, only one participant was incongruent by more than one risk categorisation (one participant scored ‘high and harmful risk’ on the AUDIT but ‘low risk’ on the ASSIST). The WHO outlines similar interventions for low, hazardous, high/harmful risk drinking on the AUDIT with that of low, moderate and high-risk drinking on the ASSIST. Thus, despite questions differing between the measures, the high degree of overlap with regard to these categorisations should, clinically speaking, reflect similar outcomes for patients if screening tools are followed as advised. Paired with the additional information provided by the ASSIST – in particular, the risky use of tobacco and cannabis – this early work suggests that the ASSIST may help to streamline the clinical interview by capturing a more complete picture of substance use without compromising information on a patient’s pattern of drinking.

This study highlights that substance use, and in particular alcohol use, is a factor in the clinical picture of Tanzanians with CPD and poor medication adherence. As adherence has important implications for individual, family and societal burden of disease, managing the factors that intersect with worse adherence is a logical step in disease management. The work presented in this article represents some of the first data to support this claim in the unique study population.

## Limitations and future work

This study has several limitations. Firstly, despite the study population constituting particularly vulnerable patients, likely in need of greater assistance and treatment resources, they represent a fairly specific subset of those in Tanzania with CPD. All the participants had recently been hospitalised and all had reported significant troubles adhering to their medications. Thus, although a potentially high-risk group, their pattern of substance use and unique distribution of attitudes towards adherence and psychiatric symptoms may not generalise to others with CPD in Tanzania.

Secondly, this study is limited in commenting on what disease management might include. What has been learnt about patterns of substance use and attitudes towards adherence, along with known interventions and previous research, must be translated into a tangible, individualised and comprehensive treatment plan. This should include assessing attitudes and willingness of Tanzanians with comorbid CPD and substance abuse to engage in treatment specific to substance use. Future work is needed in order to create substance abuse interventions that are appropriate to the unique sociocultural context experienced by those living with CPD in Tanzania.

There are limitations inherent in the sample size of the study as well. For example, comparisons were only made between low and moderate to high risk alcohol use despite the AUDIT outlining four categories of risk regarding alcohol consumption, given the small number of participants in some of the risk groups. Furthermore, this study is limited in terms of the number of female participants, particularly those at high risk of alcohol abuse. Although consistent with low rates of use seen nationally, it prevents interpretation of how alcohol use impacts treatment adherence specific to women with CPD in Tanzania.

Finally, many measures used in this study have been extensively validated in western countries, but similar validation in comparable African populations is scarce. Our study offers a preliminary comparison between the ASSIST and AUDIT, suggesting that the ASSIST is sufficient in capturing risky alcohol use in Tanzanians with CPD whilst offering the opportunity to capture additional substance use. However, the relatively small sample size and exploratory nature of this study limited more complex psychometric analyses. Also, practical implications (the time it takes to administer each questionnaire, participant’s receptivity to taking the questionnaire and usability for those administering the questionnaire) were not captured in this study. Future work is necessary to strengthen the argument for the use of the ASSIST as a streamlined instrument in the clinical practice of capturing alcohol and other substance use in Tanzanians with CPD.

## Conclusion

This study highlights hazardous alcohol use and its relationship with health behaviours and outcomes in those with serious mental illness, even in areas of the world where the sociocultural context around alcohol consumption varies from that of developed countries. This study observed the nexus between treatment non-adherence in CPD and that of alcohol use. Finally, this study provides evidence to support screening for alcohol substance use with validated, brief questionnaires such as the AUDIT and ASSIST in those with CPD in SSA.
